# Microsatellite polymorphism and its association with body weight and selected morphometrics of farm red fox (*Vulpes vulpes* L.)

**DOI:** 10.1007/s13353-014-0217-x

**Published:** 2014-05-13

**Authors:** Magdalena Zatoń-Dobrowolska, Anna Mucha, Heliodor Wierzbicki, David Morrice, Magdalena Moska, Maciej Dobrowolski, Piotr Przysiecki

**Affiliations:** 1 Department of Genetics, Wroclaw University of Environmental and Life Sciences, Kozuchowska 7, 51-631 Wroclaw, Poland; 2 The Roslin Institute, University of Edinburgh, Easter Bush, Midlothian, EH25 9RG Scotland UK; 3 Institute of Animal Breeding, Wroclaw University of Environmental and Life Sciences, Wroclaw, Poland; 4 Institute of Agriculture, State School of Higher Education, Leszno, Poland

**Keywords:** Body weight, Marker assisted selection, Microsatellites, Morphometrics, Red fox

## Abstract

Polymorphism of 30 canine-derived microsatellites was studied in a group of 200 red foxes kept on 2 Polish farms. 22 out of 30 microsatellites were selected to study association between marker genotypes and body weight (BW), body length (BL), body circumference (BC), tail length (TL), ear height (EH), length of the right front limb (FRLL), length of the right rear limb (RRLL), length of the right front foot (FRFL) and length of the right rear foot (RRFL). A total of 112 alleles and 243 genotypes were found at 22 autosomal microsatellite loci. Three monomorphic loci deemed as uninformative were excluded from the study. The association between marker genotypes and the studied traits was analysed using general linear model (GLM) procedure and least squares means (LSM). Linkage disequilibrium (LD) was estimated to assess non-random association between microsatellite loci. Out of 19 microsatellites studied four markers showed no association with the studied traits, three markers had a significant effect on one trait, and another three markers had significant effect on two traits. Among ten microsatellites with significant effect on four economically important traits (BW, BL, BC, TL) four were associated with two characters: marker *FH2613* with BW and BC, marker *FH2097*withBL and BC, marker *ZUBECA6* with BW and BC, whereas marker *REN75M10* was associated with BL and TL. The strongest LD (r^2^ ranged from 0.15 to 0.33) was estimated between nine loci with significant effect on economically important traits (BW, BL, BC, TL).

## Introduction

The red fox (*Vulpes vulpes*) had a common ancestor with the domestic dog (*Canis lupus familiaris*) about 12–15 million years ago (Bardeleben et al. 2005). Both species belong to the dog family *Canidae*, a group that has a long history spanning the last 50 million years (Ostrander and Wayne 2005). However, in contrast to *Canis lupus familiaris*, the red fox kept in captivity for more than 100 years has not been fully domesticated. Evolutionary processes of the red fox kept on farms have been influenced by artificial selection. This process, controlled by humans, is focused on retaining traits regarded as desirable (Kukekova et al. 2004). As a consequence of breeding programs carried out on fox farms not only morphological, physiological and behavioural changes have taken place, but also genetic structure of the selected fox populations has been shaped according to breeders needs.

In order to understand the genetic background of the red fox selective breeding, a genome map of the fox is needed. Evolutionary proximity to the dog enables to make good use of progress in studying the canine genome to the molecular genetics of the red fox (Sidjanin et al. 2002; Zhang et al. 2002). Development of a fox map is facilitated by the known cytogenetic homologies between the dog and fox, and by the availability of high resolution canine genome maps and sequence data. Alignment of the fox meiotic map against the canine genome sequence revealed high conservation of marker order between homologous regions of the two species (Kukekova et al. 2007). However, comparative analyses performed by mapping of DNA microsatellite markers or genes in chromosomes of the red fox, arctic fox and Chinese raccoon dog revealed that the number of the FISH mapped loci is still low (Switonski et al. 2009).

It is known that a majority of the primer sequences of canine microsatellites amplify analogous sequences in the red fox (Zajac et al. 2000). Consequently, molecular tools developed from the dog genome sequencing project (Lindblad-Toh et al. 2005) are likely to be applicable to the fox-like canids. Kukekova et al. (2004) reported, that over 60 % of tested canine-derived microsatellite markers robustly amplified fox DNA were polymorphic in foxes and were thus applicable for genotyping fox pedigrees. Because of the close phylogenetic relationship between the dog and the red fox, genomic resources developed previously in the dog have proven useful in the construction of the fox meiotic linkage map (Spady and Ostrander 2007). The first meiotic linkage map of the silver fox (which is a colour variant of the red fox) has been published by Kukekova et al. (2007).

The application of the latest advances in identifying microsatellite markers in the fox genome to red fox breeding is still in its infancy. Marker assisted selection (MAS), which combines traditional genetics and molecular biology would become a valuable tool in selecting foxes for traits of interest, such as body size, fur quality, fertility, tame behaviour or disease resistance. The information from the DNA testing, combined with the observed performance records for foxes could improve the accuracy of selection and increase the possibility of identifying individuals carrying desirable traits at an earlier stage of breeding.

The genome of the dog has the most developed set of tools among the *Canidae*, including an assembled genome sequence (Lindblad-Toh et al. 2005), which is lacking for other members of this family. Close evolutionary proximity between the dog and the red fox and high conservation of marker order between homologous regions of the two species (Kukekova et al. 2011a) offers a valuable resource for association studies in the red fox.

The aim of this study was to estimate the association of 30 canine-derived microsatellites with body weight and selected morphometrics in the farm red fox.

## Material and methods

### Material

A total of 200 unrelated silver foxes (115 females and 85 males) kept on two farms (the Leszno fox farm–120 foxes, the Prochy fox farm–80 foxes) located in a region of the Leszno city (West Poland) were studied. To ensure unrelatedness of foxes the pedigree of each animal was carefully checked. Only unrelated animals originating from different litters (one fox from a litter produced by unrelated parents) were sampled for the study. The analysed traits were: body weight (BW), body length (BL, measured from the tip of nose to the base of tail), body circumference (BC, measured behind the front limbs), tail length (TL), ear height (EH), length of the right front limb (FRLL), length of the right rear limb (RRLL), length of the right front foot (FRFL) and length of the right rear foot (RRFL). The traits measurements were taken at the end of the farm season (December) after technological slaughter of animals.

### Sampling and molecular analysis

Tissue samples for DNA extraction were taken from tongues of the studied animals. Thin slices (approximately 1–2 mm) of tongues were taken *post mortem* using a razor blade. The DNA was isolated in accordance to ARK Genomic self-protocol.

Thirty microsatellite markers described and localized in the dog genome were chosen for the study (Table 1). The multiplex PCR method (based on Qiagen Multiplex PCR Kit) was used to amplify these sequences. A whole set of microsatellites was divided into four pools for multiplex analysis (Table 2). Only three markers were amplified separately. Thirty multiplex PCR cycles at annealing temperature of 55 °C were run. After multiplex PCR, markers were genotyped using automated sequencer 3730xl (Applied Biosystems), and then obtained data were analysed using GeneMapper v. 4.0 (Applied Biosystems).

**Table 1:** Canine microsatellite markers tested in the study

Marker name	Primer sequence (5’ – 3’)	Chromosomal location (Mb)^a^	Source
*REN135K06*	AATTGATTCATGACCCACTAA	CFA01/38.8	Breen et al. 2001
	GGACCTATTCTGAAGCCTAAC		
*FH2613*	AACAATGAAAAGGAATGCCA	CFA02/49.9	Breen et al. 2001
	TAATAGCTGCTTTGAAGCCTTC		
*FH2097*	CAATGTCGAATTCCATGGTG	CFA04/79.4	Breen et al. 2001
	ATGGAGCAAGATGTGTTTGTG		
*ZUBECA6*	GCCATAAGCCCCAAGCCAGCAG	CFA05/34.4	Ladon et al. 1998
	TGCCTCGTCAGCCCCTCTTTCC		
*FH2980*	CTGGTCTCCCTTCTCTCCTC	CFA03/43.2	Breen et al. 2001
	TCTGCTTGGGCTCTCTCTC		
*REN210I14*	CTGCTCTCTCCCCCAACTTA	CFA06/47.5	Breen et al. 2001
	CAGGGCCATTGGTCTAGAAA		
*FH3970*	AAGCTTGAGTTTTGATGCTTTC	CFA07/62.3	Guyon et al. 2003
	CAGTTGGTAGAAACCAAGGAAG		
*FH3241*	AGTTTTAGCCGATCTATTTGG	CFA08/2.4	Guyon et al. 2003
	TCAAGATCCTTGTTTGGTAGG		
*UOR4101*	CCTACCATGGCAAGTGCC	CFA08/36.8	Neff et al. 1999
	TTCACGGTTGTGAGATGGAG		
*FH2263*	CATGTAGAGTGATTAGTTGGTCTTT	CFA09/9.0	Breen et al. 2001
	CTGAATATCCTCTGCCCTTC		
*REN75M10*	GCTGGGGCCTCCTCTTCTTC	CFA09/24.8	Breen et al. 2001
	GGCCCCACCTCCCCAATAC		
*AHT137*	TACAGAGCTCTTAACTGGGTCC	CFA11/1.4	Holmes et al. 1995
	CCTTGCAAAGTGTCATTGCT		
*FH3713*	TTTTTGTAAGGCAGATCTAGTGC	CFA12/52.3	Guyon et al. 2003
	GAAGCCTGTTTATGATTTTTTCTC		
*FH2060*	GTTTTGAGGAAGCCTTGCTG	CFA14/31.3	Breen et al. 2001
	GAAGGAAGGGGCCAGTATTC		
*REN307J23*	TTCCAAAAATGTATGTGTGCATC	CFA15/40.6	Breen et al. 2001
	CACTTTGTGTCAGACTTCTGGTT		
*FH2295*	TCTCGGGGATAGTGTTATAACTCC	CFA15/56.2	Breen et al. 2001
	GTCAGGAAAAGGACATTTGACC		
*REN88H03*	GATGTGAAATACCGACCTTA	CFA16/70.0	Breen et al. 2001
	AGCCTGCTTCTCCCTCTG		
*FH3775*	CCATTCAACAATAAAAGGATGG	CFA17/66.9	Guyon et al. 2003
	ACTGTTTGTTAAGGCTTGCTTG		
*FH3824*	AGGAAAAATACCAAACCAGAAA	CFA18/46.1	Guyon et al. 2003
	TTTATCTCTGATTACCTCCTGCC		
*FH3771*	GGGAAGAATACTGATAAACTGGA	CFA20/52.0	Guyon et al. 2003
	TCTTTGGTAAAGTGAAAGATTCG		
*FH2312*	AAAATAATACTCATCTATATGCTGCC	CFA21/57.6	Breen et al. 2001
	ACAACATAAGAATGTGTGTCATCA		
*FH3853*	ATAGCCAAAAGGTAGAAATAATCC	CFA22/61.1	Guyon et al. 2003
	GTAAGAGGGAGCACAAGTGG		
*FH3287*	AGGAATGCAGCAAAGTGG	CFA24/66.1	Guyon et al. 2003
	AGTCCATGCACACAGAAGG		
*FH4001*	CTATGCAGGATAATACCTTGGC	CFA27/12.2	Guyon et al. 2003
	TTAAATGTATCACCAAGCTGGC		
*REN258F18*	AAACCTTTCCATGTGGGTCA	CFA28/27.9	Breen et al. 2001
	CAAATTGCCAGAGTTGGTGA		
*REN248F14*	CACCTTCGTCTTGTGCAGTT	CFA30/27.6	Breen et al. 2001
	ACTAGGCAGGGTGAGCTGAA		
*REN64E19*	TGTATTTTAATGTGGCAGTTT	CFA34/25.4	Breen et al. 2001
	GACAAGGACAGGCAATACAGT		
*REN252E18*	CAGCATTTCCTCACTTTCCC	CFA36/33.1	Breen et al. 2001
	GGGGAGATTGTGTATCGGAA		
*REN144O22*	GAGGCTTGTTTTGTTGGGAA	CFAX/135.9	Breen et al. 2001
	GGGCAGAAGTTTTTGACCCA		
*REN44K10*	CATATTGGACCTTCACAT	CFAY/5.2	Jouquand et al. 2000
	TTAACGCACAACTTCATC		

^a^Taken from (Guyon et al. 2003). The locations were calculated with the TSP/CONCORDE program. The program calculates the intermarker distances in arbitrary TSP units, and the TSP units are translated to Mb based on the known physical size of each chromosome, as determined by cytofluorimetry

**Table 2:** Characteristics of 22 microsatellite markers selected for the study

Locus	Number of alleles	Size range (bp)	Heterozygosity	Number of genotypes	Number of individuals
Pool 1					
*FH3287*	2	286–290	0.383	3	198
*FH3771*	10	303–328	0.690	19	169
*FH3970*	6	202–219	0.680	15	163
*REN75M10*	9	167–186	0.738	17	192
*REN210I14*	4	253–270	0.438	6	198
Pool 2					
*FH2097*	8	271–365	0.671	27	199
*FH2980*	7	239–276	0.753	20	199
*REN88H03*	2	125–130	0.439	3	200
*REN135K06*	5	152–163	0.671	13	199
*REN258F18*	5	318–328	0.705	13	198
Pool 3					
*AHT137*	3	141–146	0.602	6	199
*UOR4101*	1	168	0.000	1	199
*REN248F14*	1	194	0.000	1	199
Pool 4					
*FH2613*	8	160–184	0.255	13	200
*FH3241*	4	332–340	0.509	9	194
*REN64E19*	3	156–164	0.245	5	179
*REN252E18*	1	238	0.000	1	199
*REN307J23*	3	354–360	0.419	6	197
*ZUBECA6*	12	181–296	0.698	26	200
Out of pools					
*FH3713*	5	285–310	0.594	8	199
*FH3775*	3	108–116	0.419	6	188
*FH3824*	10	259–326	0.758	28	200
Mean	5.09	–	0.485	–	–
Total	112	–	–	243	–

### Statistical analyses

The association between microsatellite genotypes and the studied traits was analysed using general linear model (GLM) procedure and the following linear model:

$$ {\mathrm{y}}_{\mathrm{i}\mathrm{jkl}}=\upmu +{\mathrm{F}}_{\mathrm{i}}+{\mathrm{S}}_{\mathrm{j}}+{\mathrm{G}}_{\mathrm{k}}+{\mathrm{e}}_{\mathrm{i}\mathrm{jkl}}, $$

where:

y_ijkl_: is the studied phenotype
μ: is the overall mean
F_i_: is the ith farm effect
S_j_: is the jth sex effect
G_k_: is the kth microsatellite marker genotype effect
e_ijkl_: is the random error associated with y_ijkl_th phenotype, *N*(0, σ^2^).

The least squares means (LSM) for the studied traits in relation to microsatellite genotype were computed using ‘lsmeans’ procedure implemented in the R package. LSM were estimated only for genotypes with the frequency higher than five. Multiple comparisons of LSM were performed with the Bonferroni-Dunn test. The studied microsatellite loci were tested separately (one at a time) and the significant differences between microsatellite genotypes for each locus were indicated.

To assess non-random association between microsatellite loci linkage disequilibrium (LD) was estimated. The squared coefficient of correlation (r^2^) computed using MIDAS (Gaunt et al. 2006) was used as a measure of LD between loci.

## Results and discussion

### Microsatellites polymorphism and heterozygosity

Table 2 lists the loci producing a PCR product. Out of 30 autosomal microsatellites 22 were selected for further analysis. Four microsatellites (*REN88H03*, *FH2613*, *ZUBECA6* and *FH3824*) were observed in all studied silver foxes, while markers *FH3970* and *FH3771* were shared by the lowest number of foxes (163 and 169, respectively). A total of 112 alleles and 243 genotypes were found at 22 autosomal microsatellite loci. The number of alleles found in the studied loci ranged from one (loci *UOR4101*, *REN248F14* and *REN252E18*) to 12 (locus *ZUBECA6*) with an average of 5.09 per locus. The three monomorphic loci (all of them were found in pool 3) deemed as uninformative were excluded from further study.

The highest heterozygosity (Table 2) was found at locus *FH3824* (0.758) followed by loci *REN75M10*, *REN75M10* and *REN258F18* (0.753, 0.738 and 0.705, respectively). The lowest heterozygosity was found at loci *REN64E19* and *FH2613* (0.255 and 0.245, respectively). An average heterozygosity per locus was estimated at 0.485. Monomorphic loci as mentioned above were not considered for further study.

The microsatellite polymorphism in foxes has not often been studied. Knowing that a majority of primers used for PCR amplification of the canine microsatellites can be successfully applied in the fox genome studies (Zając *et al*. 2000), researchers started to search for the linkage of the canine-derived microsatellites in the red fox and arctic fox genomes (Klukowska et al. 2002) or to construct a genetic map of the silver fox (Kukekova et al. 2004, 2007; Spady and Ostrander 2007).

Klukowska et al. (2002) who studied 19 canine-derived microsatellites in 14 red fox families (65 offspring) and 17 arctic fox families (113 offspring) kept on a commercial farm reported high microsatellites polymorphism in the investigated animals. The number of alleles varied between two and 14 with the mean value of 6.4 alleles in the red fox, and between one and 14 with the mean value of 8.2 alleles in the arctic fox. Kukekova et al. (2004) tested 700 canine microsatellites searching for a set of markers useful to study the fox genome. In the set of selected 30 microsatellites the calculated polymorphism information content (PIC) ranged from 0.06 to 0.77. The number of alleles per locus varied from two to 12, with a mean allele number of 5.1. The results of the cited studies are comparable to those presented in Table 2.

### Association of microsatellite genotypes with the studied traits

Least squares means and their standard errors of the silver fox morphometrics and body weight for different genotypes of ten microsatellites with significant effect on four economically important traits (BW, BL, BC, TL) are presented in Table 3. The number of foxes with a given genotype ranged from five (marker *FH2097*, genotype 275/283) to 150 (marker *FH2623*, genotype 180/180). Out of 19 microsatellites studied four markers (*AHT137*, *REN258F18*, *FH3824* and *FH3287*) showed no association with the studied traits, three markers had a significant effect on one trait (*REN135K06* with BL, *FH3713* with FRFL and *FH3775* with FRLL), and another three markers had significant effect on two traits (*REN88H03* with BW and FRLL, *FH3241* with FRLL and FRFL, *REN210I14* with FRLL and RRLL). A significant effect on the highest number of traits (five out of nine studied: BL, BC, FRLL, RRLL and FRFL) was estimated for marker *FH2097*, whereas markers *REN75M10* and *FH3970* were significantly associated with four traits (BL, TL, FRLL, RRLL and TL, FRLL, RRLL, RRFL, respectively).

**Table 3:** Least squares means (LSM) and their standard errors (SE) of studied traits for different genotypes of ten microsatellites (only significant differences are shown)

Locus	Genotype	n	LSM±SE								
			BW (g)	BL (cm)	BC (cm)	TL (cm)	EH (cm)	FRLL (cm)	RRLL (cm)	FRFL (cm)	RRFL (cm)
*FH2097*	271/279	13	6210.00 ± 310.25	69.81^ab^ ± 1.21	38.00^a^ ± 0.72	39.87 ± 0.84	9.35 ± 0.18	35.12^ab^ ± 0.80	36.96^ab^ ± 0.67	12.58^a^ ± 0.26	17.69 ± 0.26
	275/283	5	7900.00 ± 500.26	74.60^a^ ± 1.95	41.20^ab^ ± 1.17	40.00 ± 1.30	9.84 ± 0.29	32.00^ab^ ± 1.28	34.00^a^ ± 1.08	12.40^ab^ ± 0.42	17.40 ± 0.42
	275/361	6	7280.00 ± 456.67	74.00^a^ ± 1.78	42.25^b^ ± 1.06	39.67 ± 1.19	9.62 ± 0.26	33.33^ab^ ± 1.17	36.50^ab^ ± 0.99	12.17^ab^ ± 0.38	17.50 ± 0.38
	279/279	52	7083.80 ± 158.20	72.01^a^ ± 0.61	39.96^ab^ ± 0.37	39.59 ± 0.42	9.35 ± 0.09	33.52^ab^ ± 0.40	35.98^a^ ± 0.34	12.59^a^ ± 0.13	17.78 ± 0.13
	279/283	19	7409.47 ± 256.63	72.97^a^ ± 1.00	41.29^b^ ± 0.60	39.16 ± 0.67	9.53 ± 0.15	33.11^ab^ ± 0.66	35.89^ab^ ± 0.56	12.58^a^ ± 0.21	18.00 ± 0.22
	279/294	10	6719.00 ± 353.74	66.60^b^ ± 1.38	41.10^ab^ ± 0.82	38.70 ± 0.92	9.27 ± 0.20	31.90^b^ ± 0.90	34.30^a^ ± 0.77	11.30^b^ ± 0.30	17.40 ± 0.30
*FH2613*	173/180	11	6027.27^a^ ± 329.43	69.64 ± 1.32	37.32^a^ ± 0.79	39.50 ± 0.87	8.96 ± 0.18	34.64^ab^ ± 0.85	36.59 ± 0.71	12.41 ± 0.29	17.59 ± 0.28
	180/180	150	7149.80^b^ ± 89.81	71.84 ± 0.36	40.46^b^ ± 0.21	39.49 ± 0.23	9.36 ± 0.05	33.32^b^ ± 0.23	35.86 ± .019	12.43 ± 0.08	17.65 ± 0.08
*REN135K06*	152/152	45	7069.32 ± 177.35	72.63^a^ ± 0.64	39.98 ± 0.41	39.98 ± 0.43	9.33 ± 0.10	33.26 ± 0.44	35.83 ± 0.37	12.28 ± 0.14	17.76 ± 0.14
	157/159	11	6438.18 ± 354.71	67.55^b^ ± 1.29	39.64 ± 0.81	39.73 ± 0.83	9.25 ± 0.20	34.36 ± 0.87	36.91 ± 0.74	12.50 ± 0.29	17.86 ± 0.29
*REN64E19*	156/162	16	6386.67^a^ ± 290.38	69.81 ± 1.10	39.41^ab^ ± 0.64	39.19 ± 0.66	9.33 ± 0.15	35.47^a^ ± 0.71	36.99 ± 0.58	12.19 ± 0.24	17.88 ± 0.24
	162/162	135	7180.75^b^ ± 97.15	71.87 ± 0.38	40.43^a^ ± 0.22	39.37 ± 0.23	9.37 ± 0.05	33.26^b^ ± 0.25	35.71 ± 0.20	12.38 ± 0.08	17.61 ± 0.08
	162/164	23	6277.83^a^ ± 234.50	69.76 ± 0.92	38.70^b^ ± 0.53	40.71 ± 0.57	9.17 ± 0.13	35.28^a^ ± 0.59	36.85 ± 0.48	12.41 ± 0.20	17.96 ± 0.20
*REN88H03*	125/125	97	7193.16^a^ ± 117.22	71.53 ± 0.44	40.53 ± 0.28	39.55 ± 0.29	9.34 ± 0.07	33.18^a^ ± 0.29	35.88 ± 0.25	12.29 ± 0.10	17.69 ± 0.10
	130/130	27	6571.48^b^ ± 219.87	70.80 ± 0.84	39.30 ± 0.52	40.26 ± 0.53	9.53 ± 0.12	35.67^b^ ± 0.54	37.09 ± 0.46	12.43 ± 0.18	17.83 ± 0.18
*ZUBECA6*	238/238	13	7313.85^abc^ ± 299.16	72.31 ± 1.22	40.42^acd^ ± 0.69	41.12 ± 0.78	9.65 ± 0.18	34.46^ab^ ± 0.76	37.04 ± 0.66	12.81 ± 0.26	18.15 ± 0.26
	238/242	40	6677.95^ad^ ± 172.72	70.83 ± 0.69	39.96^acd^ ± 0.40	39.49 ± 0.45	9.50 ± 0.10	34.91^a^ ± 0.43	36.68 ± 0.37	12.49 ± 0.15	17.98 ± 0.15
	238/246	6	8241.67^bc^ ± 440.35	72.83 ± 1.79	42.33^ad^ ± 1.02	38.83 ± 1.15	9.58 ± 0.27	32.50^ab^ ± 1.12	35.83 ± 0.97	11.83 ± 0.39	18.00 ± 0.38
	238/292	13	5850.00^ad^ ± 299.16	69.92 ± 1.22	37.19^b^ ± 0.69	40.08 ± 0.78	9.08 ± 0.18	34.54^ab^ ± 0.76	36.15 ± 0.66	12.15 ± 0.26	17.31 ± 0.26
	238/296	6	5985.00^acd^ ± 440.35	68.67 ± 1.79	37.42^bc^ ± 1.02	38.33 ± 1.15	9.28 ± 0.27	34.83^ab^ ± 1.12	35.83 ± 0.97	11.83 ± 0.39	17.42 ± 0.38
	242/242	48	7237.23^abc^ ± 157.33	72.30 ± 0.64	40.72^acd^ ± 0.37	39.63 ± 0.42	9.33 ± 0.10	33.26^ab^ ± 0.40	35.64 ± 0.35	12.46 ± 0.14	17.71 ± 0.14
	242/246	9	7388.89^abc^ ± 359.54	71.56 ± 1.46	40.33^abcd^ ± 0.83	38.78 ± 0.94	9.18 ± 0.22	33.67^ab^ ± 0.91	35.94 ± 0.79	12.50 ± 0.31	17.61 ± 0.31
	242/272	7	7671.43^abc^ ± 407.68	74.71 ± 1.66	42.43^ad^ ± 0.95	39.86 ± 1.06	9.27 ± 0.25	33.00^ab^ ± 1.03	36.14 ± 0.89	12.71 ± 0.36	18.29 ± 0.35
	242/292	10	7250.00^abcd^ ± 341.09	68.65 ± 1.39	41.90^ad^ ± 0.79	38.90 ± 0.89	9.32 ± 0.21	31.40^b^ ± 0.86	34.40 ± 0.75	12.10 ± 0.30	17.40 ± 0.29
	242/296	9	6716.67^abcd^ ± 359.54	71.33 ± 1.46	39.39^ad^ ± 0.83	40.44 ± 0.94	9.50 ± 0.22	35.61^a^ ± 0.91	37.78 ± 0.79	12.39 ± 0.31	18.00 ± 0.31
*FH3771*	303/319	14	7885.71^a^ ± 303.38	72.64 ± 1.27	42.04 ± 0.74	39.14 ± 0.74	9.21^ab^ ± 0.17	30.86^a^ ± 0.72	35.14 ± 0.62	11.86 ± 0.26	17.36 ± 0.24
	307/307	42	6715.00^b^ ± 175.16	71.13 ± 0.73	39.81 ± 0.43	39.75 ± 0.43	9.27^ab^ ± 0.10	34.68^b^ ± 0.42	36.39 ± 0.36	12.57 ± 0.15	17.85 ± 0.14
*FH3970*	208/210	9	6261.11 ± 396.89	71.28 ± 1.52	39.56 ± 0.89	42.28^a^ ± 0.86	9.61 ± 0.20	36.28^a^ ± 0.89	38.28^c^ ± 0.78	12.33 ± 0.32	18.56^a^ ± 0.31
	210/215	42	7152.62 ± 183.72	70.96 ± 0.71	40.62 ± 0.41	38.56^b^ ± 0.41	9.28 ± 0.09	32.70^b^ ± 0.41	35.52^ab^ ± 0.36	12.45 ± 0.15	17.55^ab^ ± 0.14
	215/215	34	7223.82 ± 204.20	71.74 ± 0.78	40.31 ± 0.46	39.00^b^ ± 0.45	9.23 ± 0.10	32.62^b^ ± 0.46	35.18^a^ ± 0.40	12.31 ± 0.16	17.60^ab^ ± 0.16
	215/219	17	6834.37 ± 297.67	71.41 ± 1.14	39.63 ± 0.67	38.88^b^ ± 0.65	9.31 ± 0.15	33.06^b^ ± 0.67	35.88^abc^ ± 0.59	11.84 ± 0.24	17.28^b^ ± 0.23
*REN307J23*	354/354	10	7344.44 ± 389.46	71.90 ± 1.38	40.05 ± 0.86	37.30^a^ ± 0.86	9.43 ± 0.21	32.80^a^ ± 0.91	35.40 ± 0.76	12.05^a^ ± 0.30	17.70 ± 0.29
	354/356	38	7033.16 ± 189.54	71.30 ± 0.71	39.59 ± 0.44	40.15^b^ ± 0.46	9.26 ± 0.11	33.82^ab^ ± 0.47	35.92 ± 0.39	12.37^ab^ ± 0.15	17.58 ± 0.15
*REN75M10*	178/180	13	6815.00 ± 324.78	67.85^a^ ± 1.17	40.46 ± 0.72	39.62^ab^ ± 0.76	9.30 ± 0.19	32.69^ab^ ± 0.75	34.96^ab^ ± 0.66	12.08 ± 0.27	17.39 ± 0.26
	178/184	28	6648.93 ± 212.62	71.48^ab^ ± 0.80	39.30 ± 0.49	40.89^a^ ± 0.52	9.32 ± 0.13	35.73^c^ ± 0.51	37.52^a^ ± 0.45	12.48 ± 0.18	17.95 ± 0.18
	180/180	24	7376.67 ± 229.65	71.88^ab^ ± 0.86	40.73 ± 0.53	38.25^b^ ± 0.56	9.49 ± 0.14	31.92^a^ ± 0.55	34.54^b^ ± 0.48	12.08 ± 0.20	17.50 ± 0.19
	180/182	11	7281.82 ± 339.22	74.00^b^ ± 1.28	40.91 ± 0.79	40.70^ab^ ± 0.86	9.27 ± 0.20	32.55^ab^ ± 0.81	35.46^ab^ ± 0.72	12.46 ± 0.29	17.77 ± 0.29
	182/182	6	7656.67 ± 459.30	74.83^b^ ± 1.73	42.50 ± 1.07	39.00^ab^ ± 1.12	9.37 ± 0.27	33.50^abc^ ± 1.10	36.92^ab^ ± 0.97	13.00 ± 0.39	18.00 ± 0.39

Different superscripts indicate significance at *P* < 0.05

To our knowledge association between the polymorphism of canine-derived microsatellites and traits under selection pressure in red fox farming has not yet been studied. In the fox breeding the most important traits are those of economic importance. These are usually litter size, pelt size and pelt quality characters. According to Peura et al. (2004) who carried out the study in the Finnish blue fox population the highest relative economic weight was estimated for pelt size (0.37), followed by litter size (0.31) and pelt quality (0.26). The relative economic weights derived for the Polish blue fox population, depending on the interest rate per year assumed, ranged from 0.46 to 0.48 for litter size, 0.15 for body size, 0.35 to 0.36 for fur quality and 0.02 to 0.03 for colour type (Wierzbicki et al. 2007). In the present study four out of nine traits analysed, namely BW, BL, BC and TL, can be considered as economically important. These traits are genetically correlated with pelt or body size, the characters which significantly affect pelt prices in the international trading system (Wierzbicki and Jagusiak 2006).

The analysis of association between the studied microsatellites and four economically important traits (Table 3) indicates that significant effect on BW have five markers (*FH2613*, *FH3771*, *REN64E19*, *REN88H03* and *ZUBECA6*), while the remaining three traits are significantly affected by three markers: *FH2097*, *REN135K06* and *REN75M10* are associated with BL; *FH2097*, *FH2613* and *ZUBECA6* are associated with BC; *FH3970*, *REN30J23* and *REN75M10* are associated with TL. Out of the ten microsatellites with significant effect on the four economically important traits, four were associated with two characters: marker *FH2613* was associated with BW and BC, marker *FH2097* was associated with BL and BC, marker *ZUBECA6* was associated with BW and BC, whereas marker *REN75M10* was associated with BL and TL. Thus, in further studies on implementation of MAS in red fox breeding efforts should be focused on the set of ten microsatellite loci significantly associated with traits of breeders’ interest.

In recent years most of the association studies carried out in red foxes have mainly focused on searching for genetic markers related to body weight. Skorczyk et al. (2010), who conducted the association study in the red fox, showed that the melanocortin 3 receptor (MC3R) is a promising candidate gene for body weight. The authors reported that two polymorphisms in the red fox, i.e. a silent substitution c.957A>C and c.*185C>T in the 3’-flanking sequence, showed a significant association (*P* < 0.01) with body weight.

The polymorphism of another gene encoding melanocortin receptors, melanocortin 4 receptor gene (*MC4R*), in relation to body weight of the red fox was studied by Nowacka-Woszuk et al. (2011). In the analyzed sequence the authors found three already known SNPs, two novelindels and three novel SNPs. The 11 bp indel and four SNPs segregated as two haplotypes. The comparative *in silico* search for functional sequences in the studied fragment revealed two uORFs. However, in contrast to Skorczyk et al. (2010) the authors found no significant association between polymorphic variants within a putative upstream open reading frame of the *MC4R* gene and body weight of farmed red foxes. The polymorphism of the *MC4R* gene and its potential use in animal breeding was also investigated in the Chinese raccoon dog (*Nycterute sprocyonoides procyonoides*) and the arctic fox (*Vulpes lagopus*) (Skorczyk et al. 2012).

The polymorphisms of two candidate genes, the insulin induced gene 2 (*INSIG2*) and the fat mass and obesity associated gene (*FTO*), in four species belonging to the family *Canidae* (the dog, red fox, arctic fox and Chinese raccoon dog) were studied by Grzes et al. (2011). Two synonymous SNPs, one in the *FTO* gene (−28T>C in the 5′-flanking region) and one in the *INSIG2* (10175C>T in intron 2), were used for the association studies in the red fox. The evidence was observed for their association with body weight (*FTO*, *p* < 0.08) and weight of raw skin (*INSIG2*, *p* < 0.05). These associations indicate that both genes are potential candidates for growth or adipose tissue accumulation in the red fox.

**Fig. 1:**
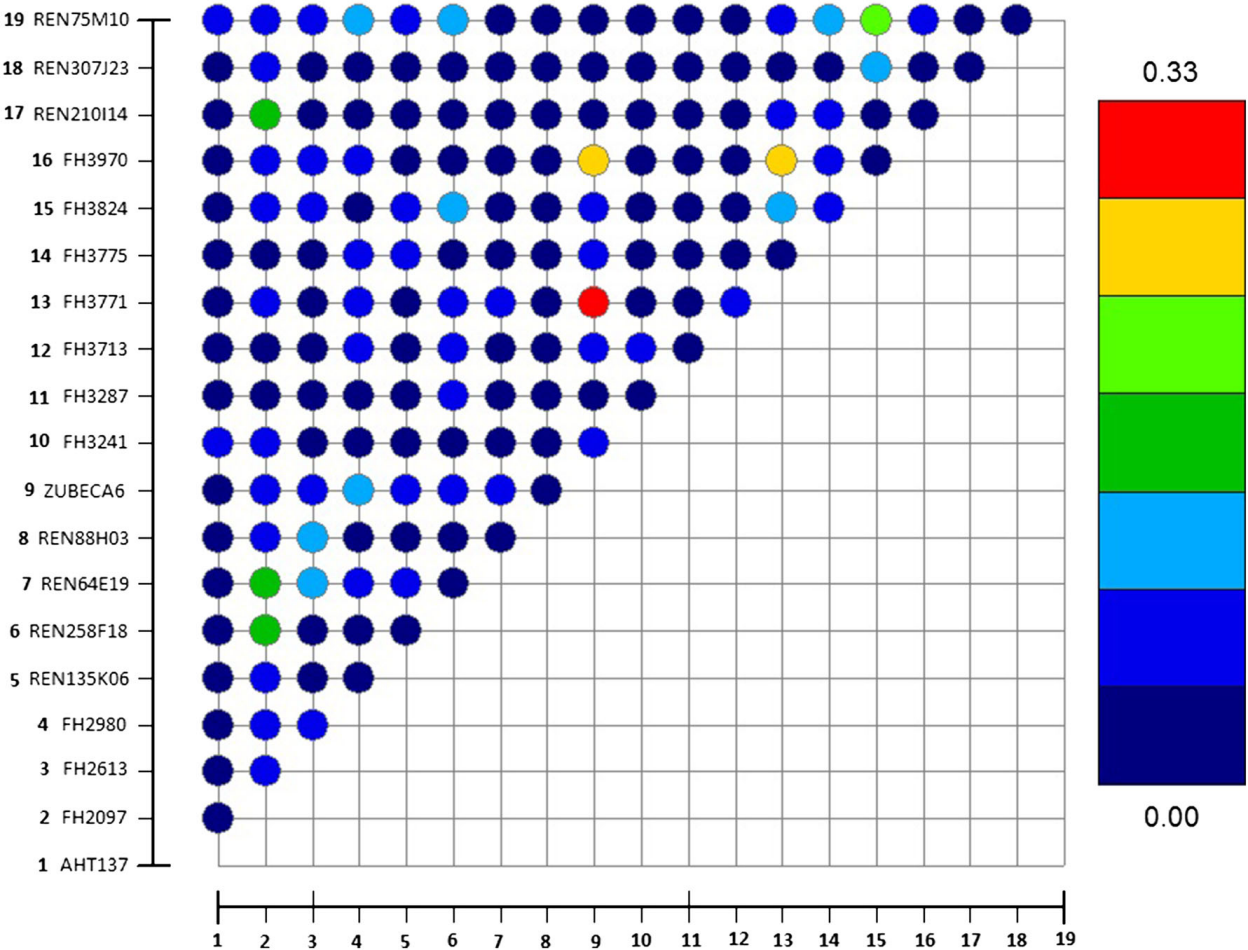
Linkage disequilibrium (LD) between the studied loci (LD was estimated only between 19 polymorphic loci)

Figure 1 shows LD between the studied marker loci. It is interesting that the highest values of r^2^ were estimated between nine loci with significant effect on economically important traits (BW, BL, BC and TL). The strongest LD was found between *ZUBECA6* and *FH3771* (*r* ^2^ = 0.33), followed by *ZUBECA6* and *FH3970* (*r* ^2^ = 0.25), *FH3771* and *FH3790* (*r* ^2^ = 0.25), *FH3824* and *REN75M10* (*r* ^2^ = 0.20). The *FH2097* microsatellite was strongly linked with three loci: *REN210I14* (*r* ^2^ = 0.16), *REN258F18* (*r* ^2^ = 0.16) and *REN64E19* (*r* ^2^ = 0.15). The pattern of LD found between the studied microsatellites may indicate that many decades of artificial selection towards genetic improvement of economically important traits in the red fox, favoured those marker loci which were tightly associated with preferred phenotypes. This strong footprint of selection in regions of the genome that are important for controlling important traits, opens up opportunities to successfully utilize LD-markers in MAS, which may markedly improve genetic progress in red fox farming.

A similar conclusion was drawn by Boyko et al. (2010) who generated a high density map of canine genetic variation by genotyping 915 dogs from 80 domestic dog breeds, 83 wild canids, and ten outbred African shelter dogs across 60,968 single-nucleotide polymorphisms (SNPs). The authors identify 51 regions of the dog genome associated with phenotypic variation among breeds in 57 traits, and concluded that artificial selection has played an important role in altering the genetic architecture of key traits in the studied canids. Identification of 44 chromosomal regions (associated, among others, with body size and ear morphology) in 46 breeds of the dog, bearing signatures of strong human-directed selection was also reported by Vaysse et al. (2011).

A closer look at the canine chromosomal regions where the studied microsatellite markers are located reveals a number of genes positioned in close distance to the markers (Guyon et al. 2003). In Table 4 the studied markers and possible candidate genes located nearby (±5 Mb) are presented. In the close proximity of eight markers located on eight canine chromosomes, 11 possible candidate genes are positioned. The selected genes are directly or indirectly involved in processes underlying behaviour (e.g. *HTR1B*), body weight (e.g. *GHR*), formation of hair structure and fur coat (e.g. *KRT17*).

**Table 4:** Description of chromosomal regions with studied markers and possible candidate genes located nearby (±5 Mb)

Chromosome	Marker and its location	Gene and its location	Gene function
CFA02	*FH2613* (49.9 Mb)	*FGF1* (46.5 Mb)	Fibroblast growth factor 1 (acidic). The protein encoded by this gene is a member of the fibroblast growth factor (*FGF*) family. *FGF* family members possess broad mitogenic and cell survival activities, and are involved in a variety of biological processes, including embryonic development, cell growth, morphogenesis, tissue repair
		*NR3C1* (47 Mb)	Nuclear receptor subfamily 3, group C, member 1, which can function both as a transcription factor that binds to glucocorticoid response elements in the promoters of glucocorticoid responsive genes to activate their transcription, and as a regulator of other transcription factors. Can act as a coactivator for STAT5-dependent transcription upon growth hormone stimulation and could reveal an essential role of hepatic GR in the control of body growth
CFA04	*FH2097* (79.4 Mb)	*GHR* (78.1 Mb)	Growth hormone receptor encodes a member of the type I cytokine receptor family, which is a transmembrane receptor for growth hormone. Function: receptor for pituitary gland growth hormone involved in regulating postnatal body growth
CFA08	*FH3241* (2.4 Mb)	*MMP14* (5.8 Mb)	Matrix metallopeptidase 14 (membrane-inserted) the protein encoded by this gene is a member of the membrane-type MMP (MT-MMP) subfamily. Acts as a positive regulator of cell growth and migration via activation of MMP15
CFA09	*REN75M10* (24.8 Mb)	*KRT17* (23 Mb)	Keratin 17 encodes the type I intermediate filament chain keratin 17, expressed in nail bed, hair follicle, sebaceous glands and other epidermal appendages. Required for the correct growth of hair follicles, in particular for the persistence of the anagen (growth) state. Modulates the function of TNF-alpha in the specific context of hair cycling
		*KFT9* (23.8 Mb)	Keratin 9, cytokeratin, encodes the type I keratin 9, an intermediate filament chain expressed only in the terminally differentiated epidermis of palms and soles. Plays a role in keratin filament assembly
		*COL1A1* (28.9 Mb)	Collagen, type I, alpha 1 encodes the pro-alpha1 chains of type I collagen whose triple helix comprises two alpha1 chains and one alpha2 chain. Type I is a fibril-forming collagen found in most connective tissues and is abundant in bone, cornea, dermis and tendon
CFA12	*FH3713* (52.3 Mb)	*HTR1B* (57.1 Mb)	5-Hydroxytryptamine (serotonin) receptor 1B, G protein-coupled 1, previously known as 5-HT1D receptors, are primarily located in the basal ganglia, striatum, hippocampus and vascular smooth muscle. 5-HT1B receptors play a role in appetite control, sexual behaviour, aggression and anxiety
CFA15	*REN307J23* (40.6 Mb)	*MYF5* (41.2 Mb)	Miogenic factor 5, involved in muscle differentiation (myogenic factor). Induces fibroblasts to differentiate into myoblasts
CFA20	*FH3771* (52 Mb)	*DAG1* (47.8 Mb)	Dystroglycan 1 (dystrophin-associated glycoprotein 1). Dystroglycan is a laminin binding component of the dystrophin-glycoprotein complex which provides a linkage between the subsarcolemmal cytoskeleton and the extracellular matrix. Dystroglycan 1 is a candidate gene for the site of the mutation in autosomal recessive muscular dystrophies
CFA34	*REN64E19* (25.4 Mb)	*SST* (28.9 Mb)	Somatostatin hormone. This hormone is an important regulator of the endocrine system through its interactions with pituitary growth hormone, thyroid stimulating hormone and most hormones of the gastrointestinal tract

Source: Guyon et al. (2003)

If the marker loci were located in chromosomal regions near the selected genes they might also create a similar genetic architecture and plausibly play a similar role in the red fox genome. However, to elucidate this the population-based genome screens together with candidate genes studies are needed.

In a group of the nine studied traits, five (EH, FRLL, RRLL, FRFL and RRFL) can be considered less important in selective breeding of foxes. These traits do not directly and significantly influence efficiency of fur production, thus the genetic improvement of these traits is not included in the breeding goal in fur farming (Wierzbicki et al. 2006). However, for evolutionary geneticists the morphological changes associated with domestication are of great interest. Domesticated animals or those being domesticated can be easily distinguished from their wild relatives by skull shape and other skeletal and morphological features (Wayne 2001). Preliminary results confirming significant differences in morfometrics between farm silver foxes and wild red foxes were reported by Zatoń-Dobrowolska et al. (2012). Development of a fox genetic map and the mapping morphological phenotypes segregating in selected red fox populations may provide insight into the mechanisms underlying the red fox evolution and domestication (Kukekova et al. 2004, 2011b).

## Conclusions

The present study confirms that canine-derived microsatellites can be successfully applied in red fox breeding. MAS using a set of selected LD-markers with significant effects on economically important traits may increase genetic progress, and thus profitability of fur farming. Further studies, including the candidate gene approach are needed to fully exploit possibilities of MAS in shaping the genetic structure of the farm red fox populations.
